# The effects of contemporaneous peer punishment on cooperation with the future

**DOI:** 10.1038/s41467-020-15661-7

**Published:** 2020-04-14

**Authors:** Johannes Lohse, Israel Waichman

**Affiliations:** 1 Department of Economics, University of Birmingham, Birmingham, B15 2TT UK; 2 Bard College Berlin, Platanenstraße 24, 13156 Berlin, Germany

**Keywords:** Psychology and behaviour, Decision making, Economics, Interdisciplinary studies

## Abstract

We use a laboratory version of the intergenerational goods game (IGG) to investigate whether peer punishment facilitates the successful provision of multigenerational public goods. In our experiment, groups (generations) decide sequentially about the provision of a multigenerational public good through the voluntary contributions of their members. Successful provision requires that contributions meet a threshold and exclusively benefits members of future generations. Provision costs are borne only by the current generation. We compare a baseline condition without a punishment institution to a treatment condition where peer punishment can be inflicted exclusively on members of the same generation but not on members of past or future generations. We find that without punishment the likelihood of reaching the contribution threshold is low and that making punishment available within a generation is partially successful in sustaining cooperation in a succession of multiple generations.

## Abstract (web summary)

Little is known about decentralized institutions that could facilitate cooperation for the sake of future generations. Here, the authors show that allowing for peer punishment within a generation is only partially successful in facilitating cooperation for the sake of later generations.

## Introduction

Humans’ unique ability to cooperate with strangers despite defection being in their material self-interest poses a major puzzle in evolutionary biology^1–5^, psychology^6^, political science^7^, anthropology^8^, economics^9–11^ and sociology^12^. Across these disciplines, there is empirical and experimental evidence that some individuals display ‘strongly reciprocal’ or ‘altruistic’ behaviour. ‘Altruism’, in this context, refers to acts of unconditional kindness towards other individuals. ‘Strong reciprocity’ describes a set of behavioural patterns that include a tendency to engage in cooperation with strangers, to reward others’ for cooperation, and to sanction others for violating a cooperative norm, even when these behaviours are costly and provide neither present nor future material benefits^10,13,14^.

In contrast to the commonly studied case of cooperation in single-generation dilemmas, where cooperation occurs within a fixed group of individuals who each bear the consequences of their own decisions and the decisions of their fellow group members^6,9^, little is known about humans’ ability to cooperate in so-called multigenerational dilemmas. In this class of social dilemmas, cooperation exclusively benefits members of future generations. At the same time, members of future generations have no ability to respond to the actions of previous generations. Examples of multigenerational public goods include the sustainable use of natural resources, the conservation of species, the prevention of climate change, or long-term investments into education, infrastructure projects, and fundamental research. The specific incentive structure of the multigenerational dilemma raises two questions. First, to what degree does voluntary cooperation emerge in multigenerational dilemmas and second is there an institution that can facilitate the emergence of cooperation in such a setting. In particular, we focus on an institution that does not require centralized enforcement, since this type of enforcement may not be feasible for some of the arguably most important multigenerational social dilemmas, which tend to be global or transnational in scope^15–17^.

To provide an answer to these two questions we draw on the findings of an experiment that was designed to explore whether contemporary peer punishment—a decentralised institution that is known to stabilize cooperation between members of the same generation^18–21^—facilitates cooperation in a multigenerational social dilemma. Our experimental design is informed by an emerging literature, which shows that delaying the benefits from cooperation typically reduces the scope of voluntary cooperation. This is true both in single-generation settings where delayed benefits accrue to the same set of decision-makers^22–26^ and in mixed-benefit settings, in which the contribution decisions of one group benefit both current and future groups^24,27–32^. We contribute to this literature, focusing on a multigenerational setting where cooperation exclusively benefits future generations. To capture central aspects of the incentive structure of a multigenerational cooperation problem, we draw on the intergenerational goods game (IGG) that was first introduced in a seminal online study^33^. From a theoretical perspective, this stylized game provides a clean testbed for exploring the effects of punishment when the benefits of cooperation only accrue to later generations, as opposed to the commonly studied single-generation case where benefits only accrue to the current generation. This full separation of costs and benefits implies that cooperating for the sake of future generations does not lie in a decision maker’s short- or long-term material self-interest.

**Fig. 1:**
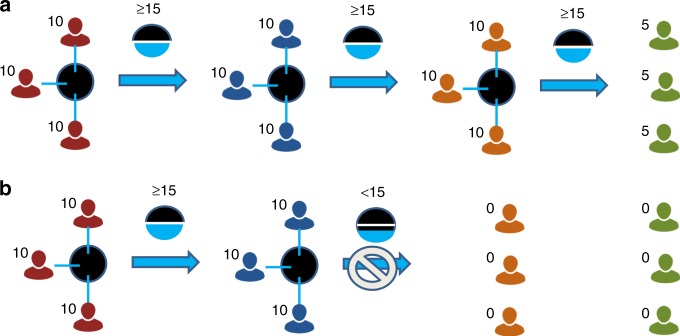
The experimental design. Each member of the first group is endowed with €10 and all three members make simultaneous contributions to the group account. **a** A case where total contributions to the group account by each group are sufficient for the game to continue. In this case, members of group 2 are also endowed with €10 each, and can likewise decide about their contributions to a group account. This process is the same for group 3. If contributions of group 3 reach the threshold, each member of group 4 receives an endowment of €5 and the game ends without any further decision being made. **b** A case where contributions of group 2 fail to reach the threshold. Hence, the game stops after each member of this group has made a decision, and members of the subsequent groups 3 and 4 are endowed with 0.

We adapt the IGG, originally proposed by ref. ^33^ for the experimental laboratory: In our implementation of the IGG, there is a succession of up to four three-player groups representing subsequent generations. The basic structure of the IGG game is shown in Fig. 1 and we describe its most important aspects below. A more detailed description of our implementation and procedures is found in the Methods section. Each experimental session (referred to as a generational sequence) consists of 12 participants who are randomly divided into four groups of three members. The experiment progresses as follows: each member of the initial group is endowed with €10 and is required to divide this amount of money between a private account and a group account. Participants earn the amount of money that remains in the private account after this decision, while contributions to the group account do not generate earnings for the participant or any other participant in his own group. If contributions to the group account sum to less than €15, the experiment ends instantly and all members of future groups earn nothing. However, if the sum of contributions to the group account is €15 or more, the game continues to the next group. Each member of the next group is endowed with €10. The same rules apply until members of Group 3 have made their decision. If the members of Group 3 contribute less than €15 to the group account, members of Group 4 earn nothing. Otherwise, members of Group 4 earn €5, but do not get to make a decision themselves. We use a contribution threshold to model situations where a minimum investment is required to provide a public good. The presence of a threshold complicates the emergence of cooperation beyond the fact that there are no material benefits from contributing to the group account. Even individuals who would otherwise be willing to provide the public good may be discouraged from contributing to its provision if they believe that the threshold cannot be met. In our setting, the successful provision of a multigenerational good hence rests on two central ingredients: a willingness to provide the public good and successful coordination on a cooperative outcome.

Our baseline implementation of the IGG shares central features with the baseline condition of ref. ^33^, such that it can serve as a conceptual replication of their design. However, we made several adaptations that facilitate a transfer of their design from an online environment to the experimental laboratory. The most important changes include a reduction of the number of members per group from 5 to 3, group members being informed about their position in the generational sequence, a fixed number of groups, higher stakes, and a framing of contributions in terms of “giving” to the group account instead of “taking” from the group account.

In their study of centralized institutions, ref. ^33^ show that median voting can overturn the adverse incentive structure of the IGG. Successful cooperation through voting is supported by the following mechanism: as long as each generation consists of a majority of cooperators, median voting can force a minority of free-riders to contribute to the public good and thereby sustain the game. Crucially, however, this version of median voting depends on the centralized enforcement of the majority outcome and hence is infeasible in those situations of interest where such centralized enforcement power does not exist^34^. Because of the global scope of some of the most important multigenerational public goods, we focus our investigation on peer punishment—an institution that does not require centralized enforcement.

For this reason, we study the effectiveness of peer punishment as one decentralized institution that has been found to be particularly effective in facilitating and maintaining cooperation in single-generation settings^18–21^. Yet, it is unclear whether peer punishment would facilitate cooperation in a multigenerational setting. One plausible impediment to the effectiveness of punishment in multigenerational settings is that it can only be inflicted on members of the same generation but not across (past or future) generations. This temporal restriction implies that those who are materially affected by a lack of cooperation cannot make use of the punishment institution. To understand how this temporal restriction affects the effectiveness of a punishment institution, we conduct a treatment which is identical to the baseline condition we describe above apart from one difference: after group members submit their contribution decisions and observe the decisions of their group members, they have the option to reduce (for a cost) the earnings of each of their contemporaneous group’s members by up to €6.

Punishment in our experiment thus shares both features of ‘second-party punishment’^18,19^ in the sense that individuals can only punish their own group members, and ‘third-party punishment’^35^, as the earnings of those deciding about punishment are not affected by the contribution decisions of those whom they can punish. The willingness to inflict second-party punishment in single-generation public good experiments is typically attributed to individuals’ desire to punish non-cooperators who have contributed less than their group members^36–39^. Little is known about the effects of (single-generation) second-party punishment when there is a contribution threshold. There is, however, evidence from single-generation experiments indicating that third-parties are willing to punish non-cooperators despite bearing no monetary consequences from their decision, although third-party punishment, typically interpreted as an expression of strong reciprocity, is found to be less severe and effective than second-party punishment^35,40,41^.

In sum, we conduct a laboratory version of the IGG in which we randomize participants to a no-punishment (baseline) and a punishment (treatment) condition. By analysing behaviour in the baseline condition we revisit the question of whether cooperation can be maintained in a multi-generation dilemma, where members of the current generation bear the full cost of cooperation while benefits only materialize for members of subsequent generations. By comparing behaviour between the baseline and the treatment condition we answer whether the availability of a peer punishment institution within a generation affects the prospect of cooperation emerging across generations.

Before we turn to the results, we will use concepts from game theory to provide some simple benchmark predictions for both the no-punishment (baseline) and the punishment (treatment) conditions. These predictions reflect the incentive structure of the IGG and depend on assumptions regarding contribution motives. For the baseline, the most basic prediction emerges under the assumption that group members are only motivated by maximizing their own experimental earnings (henceforth money maximizers). Since contributing to the group account is costly and reaching the threshold exclusively benefits future generations, money maximizers in each generation have a dominant strategy to keep the full endowment in their private account. In a (non-cooperative) subgame perfect equilibrium all group member follow this dominant strategy and, as a consequence, the threshold is not met, ending the game after the first group. In this equilibrium, total earnings available within a generational sequence are €30 as only the members of the first generation receive an endowment. This outcome is highly unequal; the three members of the first generation earn €10, whereas the remaining nine participants of the subsequent three generations earn €0.

Different predictions, including some in which the threshold is reached, emerge under the assumption that not all individuals are acting as money maximizers. Such an alternative assumption reflects a large body of evidence from single-generation social dilemma experiments showing that the behaviour of some individuals is consistent with altruism as well as a number of motives underlying strong reciprocity^10,13,14^. One motive that could underlie positive contributions in our setting is that participants in later generations could contribute to the group account because of a desire to adhere to a norm of (intergenerational) strong reciprocity^42^. Since group members in generations 2 or 3 receive their own endowment because of the contributions made by previous generations, cooperation might be seen as the prevailing social norm. More generally, motives such as altruism and those underlying strong reciprocity give rise to a number of conceivable (cooperative) equilibria in which individual contributions add up to the exact threshold amount. One particular cooperative outcome occurs when each group member contributes exactly €5. In this outcome, total earnings within a generational sequence would be maximal (€60) and earnings would be equal across participants (i.e. each of the 12 participants earn €5).

However, the absence of money maximizers from a group is a necessary but not a sufficient condition for reaching the threshold. Even under the assumption that all group members would principally want to benefit future groups through their contributions, a (non-cooperative) equilibrium, in which group members do not contribute, can still emerge. This equilibrium would arise if group members are (correctly or incorrectly) pessimistic that the threshold can be met: Even group members who desire to benefit future generations, will refrain from contributing if they believe that the threshold cannot be met because they assume that other group members will not contribute sufficient amounts.

We next turn to benchmark predictions for the punishment condition. Under the assumption that group members are money maximizers, the theoretical prediction for the subgame perfect equilibrium in the treatment condition is the same as in the baseline: contributions in each generation are zero, there is no punishment, and cooperation breaks down in the initial generation. This prediction reflects that money maximizers receive no present or future material benefits from punishing and hence they are not willing to incur a cost to punish their group members. In other words, punishment constitutes a second-order public good that will not be supplied by money maximizers. Lifting the assumption that all group members act as money maximizers affects predictions for the punishment condition and raises the possibility that one of the cooperative equilibria described above can be reached. The availability of punishment could either discipline potential non-cooperators (those who would otherwise contribute less than €5 to the group account) or, due to the threshold nature of the IGG, enhance cooperation by affecting the beliefs of potential cooperators (those who would be in principle willing to contribute €5 or more to the group account) that the threshold can be met.

The only way in which contemporary punishment can discipline non-cooperators in our setting is through deterrence (i.e. their anticipation to be punished for low contributions). Non-cooperators may expect to be punished if they believe that other group members would be willing to bear a personal cost in response to their unfair treatment of later generations, to the unequal outcome created by their decision, or to the fact that they have not cooperated while other group members have. In contrast, non-cooperators may expect not to be punished, in which case punishment would not deter decision-makers from non-cooperation. In an IGG, the decisions of group members do not affect the earnings of other group members within the same generation. Thus, if lost earnings were the main motive for inflicting costly punishment, non-cooperators would not expect to be punished. Moreover, even if they assume that their group members are acting out of purely altruistic motives, non-cooperators will not expect to be punished, since a pure altruist would not make use of a punishment opportunity that merely reduces the earnings of a non-cooperator without providing benefits to current or future group members. There is no guidance from previous studies on how effective deterrence would be in a multigenerational setting. Instead, turning to evidence from single-generation experiments, where non-cooperation also reduces the earnings of (contemporaneous) group members, deterrence alone has been found to be sufficient to facilitate cooperation in a number of instances^37,38^.

Our experimental findings can be summarized as follows: In the baseline condition without punishment the likelihood of meeting the contribution threshold is low and the sequence of generations does never progress to the final generation. The propensity of reaching the threshold is low despite a majority of participants acting cooperatively. The availability of peer punishment in the treatment condition more than doubles the likelihood that later generations are reached. However, even with punishment, cooperation is not sufficient to fully sustain cooperation over multiple generations.

## Results

### Sustained generations

**Fig. 2:**
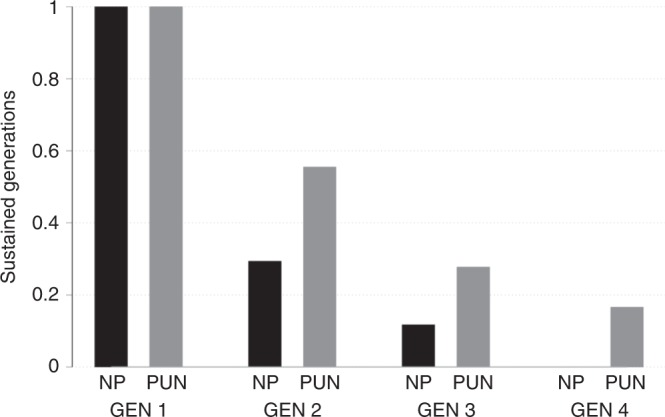
Sustained generations in the no-punishment (NP: black bars) and punishment (PUN: grey bars) conditions. The availability of a punishment institution more than doubles the average level of sustained generations across all generations. Yet, it does not preserve full cooperation across multiple generations. In the No-punishment condition, 17 groups started in GEN1, and 5, 2, and 0 groups continued to GEN2, GEN3, and GEN4. In the punishment condition 18 groups started in GEN1, and 10, and 5, and 3 groups continued to GEN2, GEN3, and GEN4.

The black bars in Fig. 2 display the percentage of sustained generations in the baseline condition (i.e. the percentage of groups that get to make an active decision in a session per generation). This figure indicates that in only 29% of the sessions without a punishment institution the game progresses to the second generation, and in a total of 12% of sessions it reaches the third generation; in none of the sessions the game reaches the final generation. This result is strikingly close to that of ref. ^33^ (sustained generations in their study: Gen. 2: 22%; Gen. 3: 11%; Gen. 4: 0%). Hence, despite the design modifications we discuss above, our laboratory version of the IGG yields virtually the same result as the baseline of the original IGG experiment^33^ and of its direct replication^43^: voluntary contributions are not sufficient to stabilize cooperation across multiple generations.

Next, answering the core question of this study, we investigate the effect of the punishment institution on the percentage of sustained generations. We do so by comparing the average level of sustained generations illustrated by the grey bars (punishment condition) and the black bars (no-punishment condition) in Fig. 2. Aggregating across the different positions in the generational sequence (i.e. across the different groups in a session), the availability of punishment increases the percentage of sustained generations by a factor of 2.36. That is 33% of all potential future generations (GEN2–GEN4) in the punishment treatment are sustained, while without punishment this is only the case for 14% of all potential future generations, a significant difference (Group-Level: Chi2-test, Chi2-statistic = 5.559, *p* = 0.018, *n* = 105). In a generation-by-generation comparison a similar picture emerges; in 29% of sessions without punishment generation 2 is reached. This rate nearly doubles to 56% in sessions with punishment. For the third generation, the effect is even larger; 28% of groups receive an endowment under punishment compared with 12% without punishment. In sessions without punishment, the final generation never receives an endowment compared with 17% of sessions with punishment.

Comparing the likelihood that a given generation is sustained conditional on reaching the previous generation controls for persistence effects. We observe a higher share of sustained generations in the punishment condition. More precisely, the conditional likelihood that a generation is sustained across all later generations (2–4) is larger in the punishment (56%) than in the non-punishment (29%) sessions (Group-Level: Chi2-test, Chi2-statistic = 3.635, *p* = 0.057, *n* = 57). The likelihood of reaching generation 3 given that generation 2 has been reached is 50% in the punishment sessions but only 40% in the sessions without punishment. This effect is larger for generation 4. Here the conditional probability is 60% in the punishment treatments but 0% without punishment. These results are further corroborated by a set of probit regressions shown in Supplementary Table 1. The availability of a punishment institution increases the likelihood of sustaining later groups. Quantitatively this effect is large, as it almost doubles the rate at which cooperation can be sustained. Yet, punishment is not sufficiently efficacious to sustain cooperation at high levels across multiple generations.

### Contribution behaviour

**Fig. 3:**
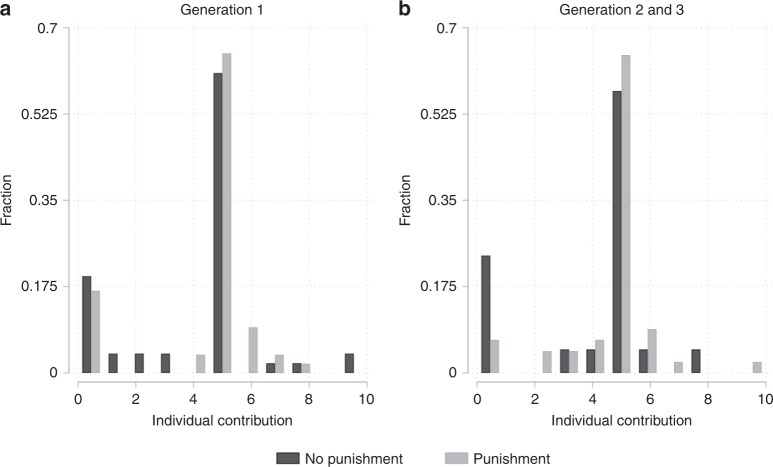
Individual contributions in the no-punishment and punishment conditions. **a** Contribution decisions of the initial group (a total 51 and 54 decisions in the no-punishment and punishment conditions, respectively). **b** Contribution decisions of subsequent groups (a total 21 and 45 decisions in the no-punishment and punishment conditions, respectively).

We next ask if and how the presence of a punishment institution affects individual contribution behaviour. The black bars in Fig. 3 show contributions of the initial group (Fig. 3a) and of the subsequent groups (Fig. 3b). This figure illustrates remarkably high levels of positive contributions in the baseline condition, indicating that a majority of participants tries to coordinate on reaching the threshold. Specifically, (i) a large majority of participants contribute a positive amount to the group account, (ii) more than half of the participants contribute exactly €5 and (iii) the contribution average does not differ significantly between earlier and later groups with slightly higher contributions in generation 2 and lower contributions in generation 3 (1: €3.96; 2: €4.40; 3: €2.50). The high percentage of participants contributing an amount of €5 or more (68%) is virtually the same as in ref. ^33^.

The high level of positive contributions in the baseline condition of our experiment is remarkable for two reasons. First, it indicates that a majority of participants in the baseline condition display behaviour that conforms with the definitions of strong reciprocity and altruism described in the introduction. Second, it indicates that this majority is also sufficiently optimistic that the threshold can be reached as to display cooperative behaviour. In contrast, only a minority of participants displays behaviour (21%) that is consistent with money-maximizing motives or low expectations that the threshold can be met. This minority of non-cooperators is, however, sufficient to hamper the rate of sustained generations and causes the complete breakdown of cooperation over multiple generations, as illustrated in Fig. 2. To improve on this low baseline rate of sustained future generations, a punishment institution must affect individual contribution patterns such that it increases a group’s propensity to meet the threshold (i.e. by changing the contribution behaviour of those who would otherwise not contribute). The comparison of the grey bars (punishment condition) with the black bars (no-punishment condition) of Fig. 3 illustrates that the shift towards cooperative contribution levels of €5 or more is relatively minor. This is especially evident with respect to the initial generation (Fig. 3a) but also with respect to later generations (Fig. 3b).

Across all active generations, average contributions are 14% larger in the punishment than in the no-punishment condition (4.47 vs. 3.93, M.W. rank-sum-test, z-statistics = 1.822, *p* = 0.069, *n* = 171). In addition, the rate of zero contributions is nearly half the size in the punishment compared with the no-punishment condition (12% vs. 21%, Chi2-test, Chi2-statistic = 2.379, *p* = 0.123, *n* = 171). Most importantly, classifying participants with a contribution of €5 or more as cooperators, we find that the percentage of cooperators is 11 points larger in the punishment condition than in the no-punishment condition (79% vs. 68%, Chi2-test, Chi2-statistic = 2.512, *p* = 0.113, *n* = 171). The lack of significant differences in average contributions between the punishment and the no-punishment condition is further corroborated by a set of regression models displayed in Supplementary Table 2. One reason why punishment may affect contributions only insignificantly is a low deterrence effect due to low expectations that non-cooperation will be punished. In later generations, the deterrence effect of punishment appears to be larger and has a strong and statistically significant effect on the incidence of zero contributions (7% vs. 23%, Chi2-test, Chi2-statistic = 3.950, *p* = 0.047, *n* = 66). This may reflect that in later generations a norm of (intergenerational) reciprocity is more salient and could suggest to participants that they may be punished more severely for not contributing to the public good^42^. The observation that relatively small changes in individual contribution patterns are sufficient to lead to a significantly higher rate of sustained generations finds additional support in a bootstrapping exercise in which we create 10,000 hypothetical generation sequences for each condition by randomly sampling from participants’ contribution decisions (see Supplementary Note 2)^33^.

### Determinants of receiving punishment

We close by investigating the determinants of receiving punishment. This could shed some light on prevailing contribution norms in the IGG. Punishment occurs almost exclusively in groups that do not reach the threshold, where an average of 0.51 punishment points is inflicted (of a maximum 4 points). Breaking this average further down by generation, there is more punishment in unsuccessful first-generation groups (0.67), where missing the threshold inflicts most harm on future generations, than in the second generation (0.47). In the third generation where there is no gain from cooperation in terms of total earnings and where the harm from missing the threshold is thus smaller, we observe no punishment. Punishment is significantly different from zero in the first and second generation (*p* < 0.01 and *p* < 0.05, one-sided sign rank test). Similarly, 42% of the participants in the first generation and 33% of the participants in the second generation make use of the punishment opportunity when the threshold is missed.

We further explore the determinants of receiving punishment via regression analysis (Table 1). Model (1) only includes members of Group 1 and shows that individuals within a group that has not reached the threshold, and individuals contributing on average less than their remaining two group members receive significantly more punishment. Model (3) finds similar results when analysing data from all generations. The significant interaction term in models (2) and (4) furthermore shows that deviations from the norm are less severely punished when the group reaches the threshold. Finally, the amount contributed does not itself have a significant effect on the amount of punishment received. The finding that punishment is systematically directed towards those who deviate from the average contribution in a group is in line with previous studies on peer punishment in single-generation settings^18,19^. Moreover, in the final questionnaire we ask participants to state a reason for inflicting punishment: more than 90% state a reason that demonstrates fairness concerns towards members of later groups. This is in line with earlier second-party punishment studies in the sense that they find that punishers are mainly motivated by a desire to punish non-cooperators^36–39^. Our findings demonstrate that these punishment motives extend to members of subsequent groups. This mirrors earlier results on third-party punishment that have been taken as evidence for the existence of strong reciprocity^35^.

**Table 1:** Determinants of receiving punishment.

	Model (1)	Model (2)	Model (3)	Model (4)
Received punishment	GEN1	GEN1	GEN1–3	GEN1–3
Reached threshold (1 = yes)	−3.17** (1.21)	−3.17** (1.24)	−1.42*** (053)	−1.42*** (0.53)
Deviation from others’ average contributions	1.10** (0.47)	1.11** (0.48)	0.62*** (0.22)	0.66*** (0.23)
Threshold × deviation	–	−0.69*** (0.21)	–	−0.47* (0.25)
Contributions	0.64 (0.61)	0.64 (0.62)	−0.04 (0.28)	−0.04 (0.28)
Constant	−0.12 (2.14)	−0.12 (2.19)	1.66 (1.09)	1.66 (1.10)
Observations	54	54	99	99
R-squared	0.56	0.57	0.53	0.54

Note: OLS regression (robust standard errors in parenthesis); *p ≤ 0.1, **p ≤ 0.05 and ***p ≤ 0.01. The dependent variable “Received punishment” is the sum of punishment inflicted on the respective participant multiplied by the fine of €3. “Reached threshold” is a dummy variable valued 1 if the group contributes at least €15, and 0 otherwise. “Deviation from the others’ average contribution” is defined as the “average contribution of the other two group members minus own contribution”. “Threshold × deviation” is the interaction term of these two variables. Finally, “Contributions” refers to the amount of money transferred to the group account by the participant.

### Gains in aggregate earnings

Are there overall gains in aggregate earnings from introducing contemporaneous peer punishment? To answer this question we compare experimental earnings of participants assigned to the punishment and no-punishment conditions. Overall, we do not find that average earnings in the punishment condition differ significantly from those in the no-punishment condition (M.W. rank-sum-test, z-statistics = 1.602, *p* = 0.109, *n* = 420). While the punishment institution increases the number of sustained generations and hence the earnings from successful cooperation per session, it is costly to inflict punishment. In our setting, these punishment costs are sufficiently large to offset the gains from cooperation.

## Discussion

The ability of humans to cooperate with strangers is of major interest in anthropology, biology, economics, political science, psychology and sociology. Cooperation for the sake of future generations is a particularly strong expression of this ability. In this respect, field examples of multigenerational public goods provision suggest that cooperation for the benefit of later generations is often hard to sustain even when its absence can have large detrimental effects. A better understanding of the motivations and institutions that facilitate intergenerational cooperation is hence of momentous practical importance.

Our study investigates, whether an institution that complies with two crucial requirements (intergenerational institutions are only available within a generation and are decentralized) can stabilize cooperation across multiple generations. To this end, we conduct a behavioural experiment using a modified IGG design^33^ that offers the possibility of observing cooperation in a purely multiple-generation setting. From a theoretical vantage point, cooperation in such settings does not offer any contemporary benefits and hence can only be supported by a combination of (i) motives underlying strong reciprocity and altruism, and (ii) successful coordination on reaching a provision threshold.

Our baseline results are closely in line with those derived from a series of internet-based experiments that introduced the IGG as a tool for studying cooperation across multiple generations^33^. This similarity not only provides robustness to the finding that cooperation for the sake of future generations is hard to sustain in the absence of additional institutions. It also shows that our modified version of an IGG can be used to study similar questions in the experimental laboratory, thereby offering experimentalists a practical method for conducting IGG studies offline.

Our main findings are that while peer punishment has only a small effect on individual contributions, its availability more than doubles the likelihood that later generations are reached. Yet, even with punishment, the second generation is sustained in only 56% of all cases and only 17% of generational sequences reach the final generation. Hence, peer punishment is only partially successful in sustaining cooperation over multiple generations. A second striking finding of our study is that even without punishment a large majority of participants acts cooperatively while only a minority acts in a money-maximizing fashion. Given this high baseline level of cooperative behaviour, the availability of contemporaneous peer punishment has a relatively moderate effect on increasing the share of participants who act cooperatively. However, it harnesses the already large majority of cooperators and considerably increases the number of sustained generations by changing the behaviour of a small number of non-cooperators that would otherwise prevent a group from reaching the threshold.

Thus, in a population where a majority of individual are already acting cooperatively, the effectiveness of any institution hinges on its ability to discipline a small sub-population of non-cooperators. Previous evidence shows that one way to reach such efficiency is to force a non-cooperative minority to comply with the will of the majority^33^. Yet, there are many multigenerational problems, often those that are global in nature, where the will of the majority cannot be easily enforced in this way. Decentralized institutions like the punishment institution we study rest instead on the majority’s ability to deter the non-cooperative behaviour of a minority. This kind of deterrence may not always be credible, especially when it is costly and does not promise personal benefits in the presence or the future. Hence, one exciting path for future research on multigenerational cooperation is to investigate the effect of peer punishment in conjunction with other institutions that would make the threat to non-cooperators more credible.

## Methods

### Design

Figure 1 illustrates the game structure and procedures. Exactly 12 participants take part in each session and they are randomly divided into four groups consisting of three participants each. In the baseline condition, participants are informed that they will make (at most) one decision and that there are a total of four sequential groups. Moreover, they are informed about the position of their group in the succession of groups and learn that the allocation of participants to the groups is random. After reading a printout of the instructions (reproduced in Supplementary Note 3) and being given an opportunity to ask clarifying questions, the experiment starts. In the baseline condition, each member of Group 1 is endowed with €10. The three members of this group simultaneously face the same decision: they are required to divide their endowment between a private account and a group account. More precisely, they are required to decide how much money to allocate to the group account while the rest of the endowment automatically remains in their private account. If individual contributions to the group account sum to less than €15, the experiment ends instantly and all participants of later groups are informed that the experiment has ended and that their earning from the experimental task are zero. However, if the sum of contributions to the group account amounts to €15 or more (i.e. at least 50% of the total group endowment), the game continues and moves to the next group (Group 2). Independent from the sum of total contributions in the group account, members of Group 1 earn the respective amounts in their private accounts (i.e. the initial endowment of €10 minus their individual contribution to the group account). If the game is sustained, the same procedure applies for members of Groups 2 and 3. The experiment ends with the decision of the members of Group 3. If the contributions of Group 3’s members to the group account sum to €15 or more, then the members of Group 4 each earn €5. Otherwise, members of Group 4 earn nothing. Reaching the threshold exactly costs €15 to the current generation but provides an endowment of €30 to the subsequent generation(s). There is hence a gain in total earnings (across multiple generations) from reaching the threshold. These potential gains from contributing are largest for members of the first generation, but smaller for participants in subsequent generations (with no such gains from Group 3). Choosing €15 as a threshold value has the advantage of facilitating coordination between group members by allowing for individual contributions of €5 (i.e. 50% of the endowment) to be sufficient for reaching the threshold. Hence, it is a symmetric focal point for coordination. At the same time, individual endowments are smaller than the threshold. Hence, no single group member can unilaterally provide the public good and successful provision requires the cooperation of at least two group members.

On a session level, experimental participants are randomly allocated either to this no-punishment (baseline) condition or to a punishment (treatment) condition. In the punishment condition, the game structure is identical to that described above, except that after the contribution stage, members of the current generation have access to a costly peer punishment institution. In both the baseline and the treatment conditions, a feedback screen, shown after the contribution stage, informs participants about the individual contributions made by each member of their group (but provides no information on contributions made by members of previous groups). In the punishment condition, participants can use this screen to inflict up to two punishment points on either of the other two group members (identifiable by a number). They cannot inflict punishment on members of other (past or future) groups. We follow the seminal designs of previous punishment experiments^18,19^ and employ a fine-to-fee ratio of 3:1 (i.e. for a cost of €1 per punishment point inflicted, the punisher reduces the punishee’s earning by €3)^44,45^. Punishment can reduce a punishee’s earning to zero but not to a negative amount.

### Procedure

The experiment was conducted at two large German universities from January to August of 2017. Participants were recruited using hroot^46^. We conducted a total of 35 sessions with 12 participants each, for a total of 420 student participants. The 35 total sessions were comprised of 17 sessions (*n* = 204) of the baseline condition and 18 sessions (*n* = 216) of the punishment condition. Sessions of both types were run at both locations. Since we found no differences in contribution behaviour between the locations (overall: *p* = 0.62; punishment sessions: *p* = 0.56; no-punishment sessions: *p* = 0.99; M.W. rank-sum-test), we pool observations from both participant pools. At the end of the experiment, participants in the punishment sessions answered a short questionnaire regarding the motives behind their decisions regarding punishment. Experiments were conducted according to the guidelines provided for study procedures at the respective institutions where the data were collected. The experimental interface was computerised using z-Tree^47^.

The experimental sessions lasted between 25 and 40 min and participants’ earnings were paid in private at the end of each session (M = €7.23, SD = €2.93).

The study complies with the ethics procedures and code of scientific integrity for non-medical research set out by Heidelberg University and the Alfred-Weber-Institute (AWI). Informed consent is given by all participants at the time of registering as subject participants. Participants can discontinue the experiment at any time without negative consequences.

### Reporting summary

Further information on research design is available in the Nature Research Reporting Summary linked to this article.

## Supplementary information

Supplementary Information

Reporting Summary

## Data Availability

The experimental data that support the findings of this study is available in figshare with the identifier: https://figshare.com/s/f140d450cb6758375da7. A reporting summary for this Article is available as a Supplementary Information file.
