# Supplementary material for: The effects of contemporaneous peer punishment on cooperation with the future
Source: Nat Commun. 2020 Apr 14;11:1815. doi: 10.1038/s41467-020-15661-7 (PMC7156437; doi:10.1038/s41467-020-15661-7)
Supplement: Supplementary file 1 — Supplementary Information [file 41467_2020_15661_MOESM1_ESM.pdf]

Supplementary Information for

"The Effects of Contemporaneous Peer Punishment on Cooperation with the Future"

Joahnnes Lohse and Israel Waichman

March 2020

Supplementary Note 1: Regression Results

Supplementary Table 1: Regression results: reaching the threshold

Reaching the threshold (1=Yes)	(1) First generation only	(2) All active generations	(3) All active generations
Punishment treatment	0.68	0.66*	0.67*
	(0.44)	(0.35)	(0.35)
Group 2	-	-	-0.00
			(0.41)
Group 3	-	-	-0.15
			(0.51)
Constant	-0.54*	-0.55**	-0.54*
	(0.33)	(0.27)	(0.29)
# of observations	35	57	57
Pesudo-R^2	0.05	0.05	0.05

Note: Probit regression with observations at the group level (robust standard errors in parentheses); *p ≤ 0.1, **p ≤ 0.05 and ***p ≤ 0.01.

Supplementary Table 1 contains a set of binary Probit regression models: Model (1) considers only the first generation while models (2) and (3) include all active groups. Model (3) controls for the position in the generational sequence. The treatment dummy is positive in all models, but only marginally significant in the models including all generations (2 and 3).

Supplementary Table 2: Regression results: contributions

Contributions	(1) First generation only	(2) All active generations	(3) All active generations
Punishment treatment	0.39	0.54	0.53
	(0.45)	(0.35)	(0.36)
Group 2	-	-	0.25
			(0.38)
Group 3	-	-	-0.13
			(0.45)
University	-	-	0.02
			(0.34)
Constant	3.96***	3.93***	3.86***
	(0.35)	(0.29)	(0.58)
Observations	105	171	171
R-squared	0.01	0.02	0.02

Note: OLS regression (robust standard errors in parenthesis); *p ≤ 0.1, **p ≤ 0.05 and ***p ≤ 0.01.

Supplementary Table 2 contains a set of OLS regression models: These models estimate the treatment effect of punishment on contributions for the first generation only (1) or for all active decisions (2)-(3). Model (3) controls for the position in the generational sequence and also controls for participant pool effects. While the average treatment effect of punishment is positive in each model, it is not sufficiently large to reach statistical significance in any model. Similar results emerge when using probit models to test either the probably for free riding (contributing nothing) or for coopering (contributing at least €5 or more).

Supplementary Note 2: Simulation

This section reports on a simulation exercise that aims at testing the robustness of one of our main result. As we report in the main text of the paper, we observe a sizable aggregate effect of the punishment institution on sustained generations although the same institution has only a subtle effect on contributions at the individual level. To illustrate how individual level contributions map into the rate of sustained generations, we complement the analysis of primary data collected in our experiment with a simulation exercise. The mapping of individual-level contributions into aggregate provision levels in the IGG framework is subject to multiple sources of noise that exist independently from randomizing experimental participants to treatment conditions and groups within a session.[1] In particular, assigning participants to specific groups and placing these groups at a specific position in the generational sequence could have a strong influence on session-level outcomes i.e. the number of sustained generations. For instance, imagine a session which consists of three free-riders and nine co-operators. There is only one way of randomizing participants to groups that would lead to groups reaching the threshold until Group 4. Any other way of randomizing participants to groups within such session will lead to an earlier break down of the generational sequence. The number of actual group matchings that we observe in our dataset is far more limited than the number of all conceivable combinations due to the finite number of experimental sessions that can be possibly conducted for each treatment condition.

The simulation exercise follows the methods described in ref 1 by generating simulated generational sequences: In particular, we run a bootstrap procedure that simulates the aggregate number of sustained generations in 10,000 artificially generated generational sequences for each

of our treatment conditions. For each of these simulated generational sequences, we sample (with replacement) individual contributions for each potential decision made within these sequences from our observed experimental data. We then determine for each simulated generational sequence the point at which it would break down. Overall, this simulation lends further support our results of the considerable increase in sustained generations. As summarized in Supplementary Table 3, despite the relatively modest differences in individual contribution levels observed in our experimental data, the availability of a punishment option increases the number of sustained generations at each step of the generational sequence and these small differences ensures a higher aggregate number of sustained generations (Panel B).

Supplementary Table 3: Sustained generations based on a bootstrap simulation

	Generation 2	Generation 3	Generation 4	Overall
	Panel A: Experimental data			
No-Punishment	29%	11%	0%	13%
Punishment	56%	28%	17%	33%
Treatment Difference	26%	17%	17%	20%
	Panel B: Simulated data			
No-Punishment	37%	17%	2%	19%
Punishment	53%	27%	15%	32%
Treatment Difference	16%	10%	13%	13%

Note: This table displays the percentage of sustained generations in either the experimental data (Panel A) or of the simulated data (Panel B). “Treatment Difference” indicates the difference in survival rates between the punishment and no-punishment treatments.

Supplementary Note 3: Translated instructions

The original German version is available on request.

<u>Paper instructions (Punishment Condition)</u>[1]

Dear participant,

Thank you for participating in our experiment. For your participation, you will earn 5 Euros as a participation fee. You can earn more money. These additional earnings depend on your decisions and also on the decisions of the other participants in the room during the experiment. In the following, we will explain the task of the current experiment.

All participants receive the same instructions as you. You should read the instructions quietly and thoroughly. Please do not talk with other participants in the experiment. Should you have any question, please raise your hand and wait until we will come to your computer terminal and answer your question. In case you will talk with other participants; you will be unfortunately asked to leave the experiment. In this case, you will not earn any money. Please make sure to shut down your cell phone or turn it on silent mode.

In the decision task will not talk about Euros, but rather about Money Units (MU). The amount that you will receive for the decision tasks would be calculated and shown in MU. At the end of the experiment, the total amount (including the 5 Euros participation fee) will be converted to Euro and will be immediately and anonymously paid to you. The exchange rate between MU and Euros is:

1 MU: 1 Euro

This means that if you earned 1 MU, you will receive 1 Euro.
All your decisions are recorded anonymously, and will be only used for a scientific purpose.

The decision task

Group assignment

A total of 12 participants take part in the current experiment. These 12 participants will be randomly and anonymously divided into four groups, each group consists of three participants. The different groups will be labeled during the experiment as Group 1, Group 2, Group 3, and Group 4. A random mechanism (similar to a toss of a die) will determine if you will be assigned to Group 1, 2, 3, or 4. Once the computer program starts, you will see on your computer screen if you are assigned to Group 1, 2, 3, or 4.

Decisions

[1] The explanation for the no-punishment condition is equivalent apart from the section explaining the punishment procedures and small detail (e.g. one instead of two decisions)

In the current experiment, you will take (at most) two decisions which appear on two subsequent computer screens. The rule according to when you can make a decision depends on which group you are randomly assigned to. The decisions will be taken one group after another, starting with Group 1. This means that first all members of Group 1 simultaneously take their first decision (i.e., if you are a member of Group 1 you cannot see what decisions the members of your group have made until all group members have submitted their decisions). For this decision, each participant receives an endowment of 10 MU (a total of 30 MU per group). Then each participant can decide how much MU to keep and how much MU to deposit in a group account. MU that a participant keeps will be paid to this participant at the end of the experiment. The MU in the group account will be added up. Then each group member will be able to see how many MU the group members (labeled as participants 1, 2, and 3) have assigned individually to the group account as well as the sum of the amount assigned to this account. If the sum is **15 MU or more**, the experiment continues further. If the sum is **less than 15 MU**, the experiment ends. In this case, after the group members will make the second decision, each of them will earn 5 Euros participation fee and the amount they earn from the decision (this implies 0 additional earnings for Groups 2, 3, and 4).

After you find out how much the other members of your group have assigned to the group account, you proceed to the computer screen of the second decision task. In this decision task, you have the possibility to penalize or not members of your group. For penalizing you need to use the remaining MU that you kept (i.e., the endowment minus the amount that you assigned to the group account). You can assign to each of your group members 0, 1, or at most of 2 negative points. Each negative point that you assign costs 1 MU. You cannot assign more negative points than your remaining MU. (i.e., if you have for instance only 2 MU you can at most assign 2 negative points to one member (2 MU) or you can assign to each of the two group members one negative point (2x 1 MU)). If you are not following this rule, you will get a reminder on the computer screen. On the screen where you decide how many negative points to assign, you also see once again how many MUs each of the group members has assigned to the group account. Each negative point that you assign is multiplied by three and reduces the remaining MU of the penalized member (i.e., the group member who received the negative points). You have three possibilities:

You assign 0 negative points (the target member receive 0 negative points)
You assign 1 negative points (the target member receive 3 negative points)
You assign 2 negative points (the target member receive 6 negative points)

After your decision how many negative points to assign to each group member, you can see on your computer screen how much you have paid and how many negative points you have received. All decisions are anonymous: you cannot find out which participant in the room has assigned negative points to you or to which participant you have assigned negative points. Your payoff for the task will be as follows:

Endowment – assigned amount to the group account – assigned negative points – received negative points x 3. In cases where you earn a negative payoff, it will be set to 0 (and in any case, you will earn the participation fee).

In cases where the experiment continues (because the members of Group 1 have accumulated a sum of at least 15 MU in their group account), each member of **Group 2** receives an endowment of 10 MU and can decide how many MU he/she wants to assign to the group account of Group 2. All decisions in this stage are taken simultaneously on the first decision computer screen (i.e., a member of Group 2 cannot see what the other member have decided until all of them, including him/herself, have made their decisions). The sum assigned to the group account will again be added up. Afterwards, each member can see how many MU each member has assigned individually to the group account and how much has been assigned in total to the group account. If the amount is **15 MU or more**, the experiment continues further. If the amount is **less than 15 MU**, the experiment ends. In this case, after the group members will make the second decision, each of them will earn 5 Euros participation fee and the amount they earn from the decision (this means 0 additional earnings for Groups 3, and 4). Next, you proceed to the computer screen of the second decision task. In this decision task, you have the possibility to penalize or not members of your group (Group 2) (the decision is identical to those described above for Group 1). After your decision how many negative points to assign to each group member, you can see on your computer screen how much you have paid and how many negative points you have received. All decisions are anonymous: you cannot find out which participant in the room has assigned negative points to you or to which participant you have assigned negative points. Your payoff for the task will be as follows:

Endowment – assignment to the group account – assigned negative points – received negative points x 3. In case you earn a negative payoff, it will be set to 0 (in any case you will earn the participation fee).

In cases where the experiment continues (because the members of Group 2 have accumulated a sum of at least 15 MU in their group account), each member of **Group 3** receive an endowment of 10 MU and can decide how many MU he/she wants to assign to the group account of Group 3. All decisions in this stage are taken simultaneously on the first decision computer screen (i.e., a member of Group 3 cannot see what the other member have decided until all of them, including him/herself, have made a decision). The amounts assigned the group account will be added up. Afterwards, each member can see how many MU each member has assigned individually to the group account and how much has been assigned in total to the group account. If the amount is **15 MU or more**, the experiment continues further. If the amount is **less than 15 MU**, the experiment ends. In this case, after the group members will make their second decision, each of them will earn 5 Euros participation fee and the amount they earn from the decision (this means 0 additional earnings for Group 4). Next, you proceed to the computer screen of the second decision task. In this decision task, you have the possibility to penalize or not members of your group (Group 3) (the decision is identical to those described above for Group 1). After your decision how many negative points to assign to each group member, you can see on your computer screen how much you have paid and how many negative points you have received. All decisions are anonymous: you cannot find out which participant in the room has assigned negative points to you or to which participant you have assigned negative points. Your payoff for the task will be as follows:
Endowment – assignment to the group account – assigned negative points – received negative points x 3. In case you earn a negative payoff, it will be set to 0 (in any case you will earn the

participation fee).

In cases where the experiment continues (because the members of Group 3 accumulated a sum of at least 15 MU in their group account), each member of **Group 4** receives an additional endowment of 5 MU and the experiment ends.

At the end of the experiment, you will receive your earnings one-by-one and anonymously (5 Euros participation fee and the amount that you have earned in the decision tasks), such that no other participant can find out to which group you are belonging to or how much you have earned individually.

Supplementary Note 4: Computer screens (and translation)

The initial screen (when entering the laboratory and sitting in front of the computer terminal

Thank you for participating in our experiment.

Please read the instructions that are placed in front of you carefully.

Should you have any question, please raise your hand and wait until we will come to your computer terminal.

The experiment will commence once you press on "continue".

[Bottom right coroner "continue" button]

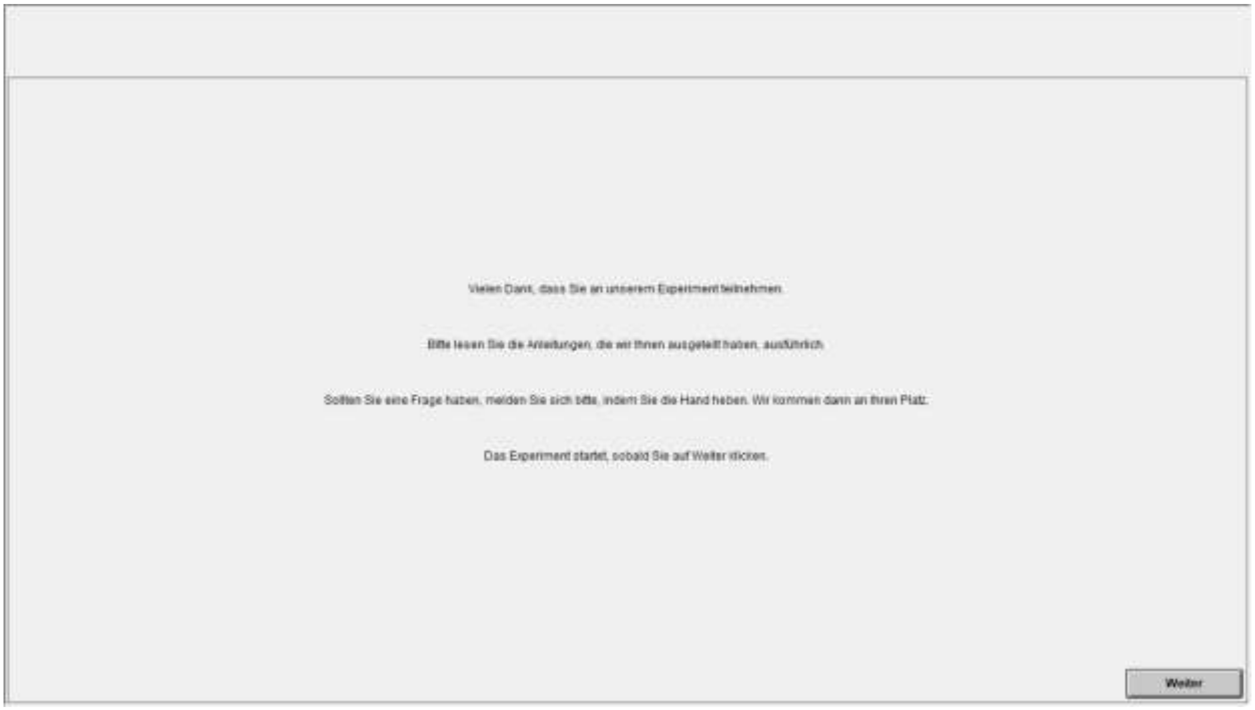

The first decision screen

For this task, you and two additional group members have received an endowment of 10 money units (MU). On this screen, you can decide how many (0-10) of your MU you want to assign to a joint group account. All MU that would be assigned to this group account will be added up. In case the sum is 15 MU or more than 15 MU, the group after you will be able to make the same decision as you. In case the sum is less than 15 MU, the decisions task will end and the next group cannot make a decision (and will not receive an endowment).

Please state here, how many MU you want to send to the group account

[Bottom right coroner "OK" button]

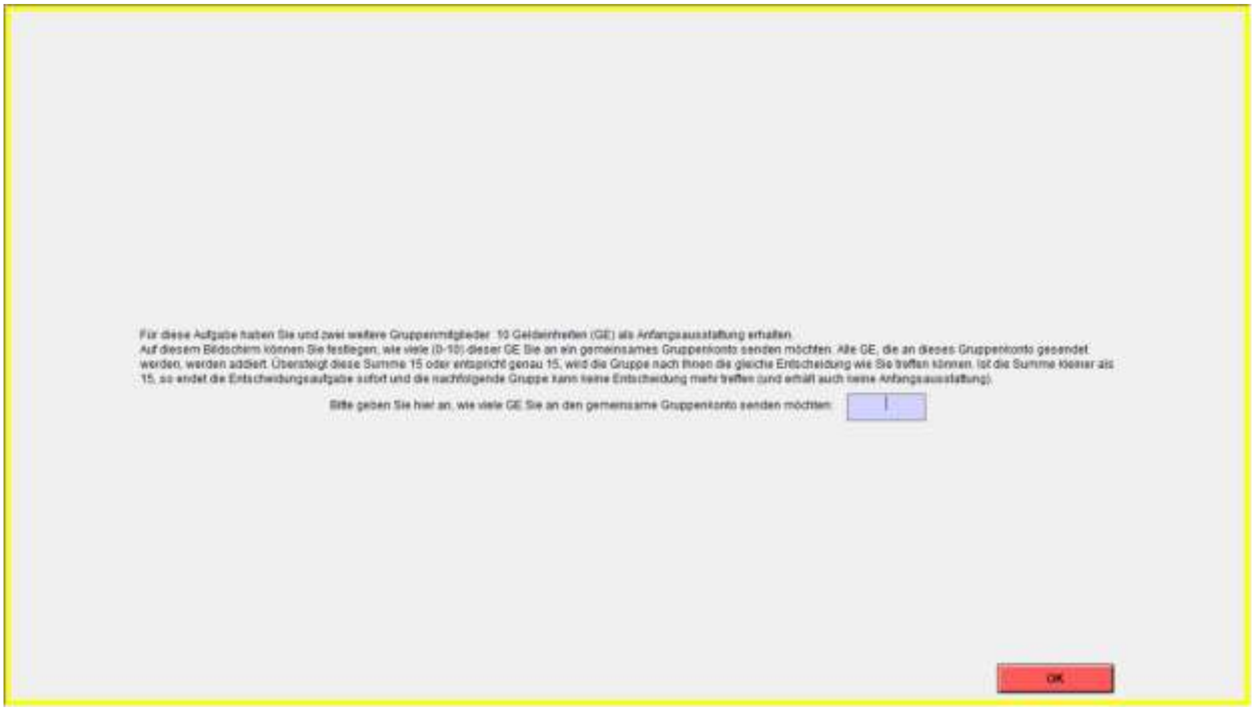

The first feedback screen

[Top panel:]

Here you are informed about the decisions of the participants in your group

You assigned amount: 7

[Middle panel:]

Player	Payment to the group account
1	5
3	6

[Bottom panel:]

Thus, there are more than 15 money units in total and, as a consequence, the next group can take part in the task

[Bottom right coroner "OK" button]

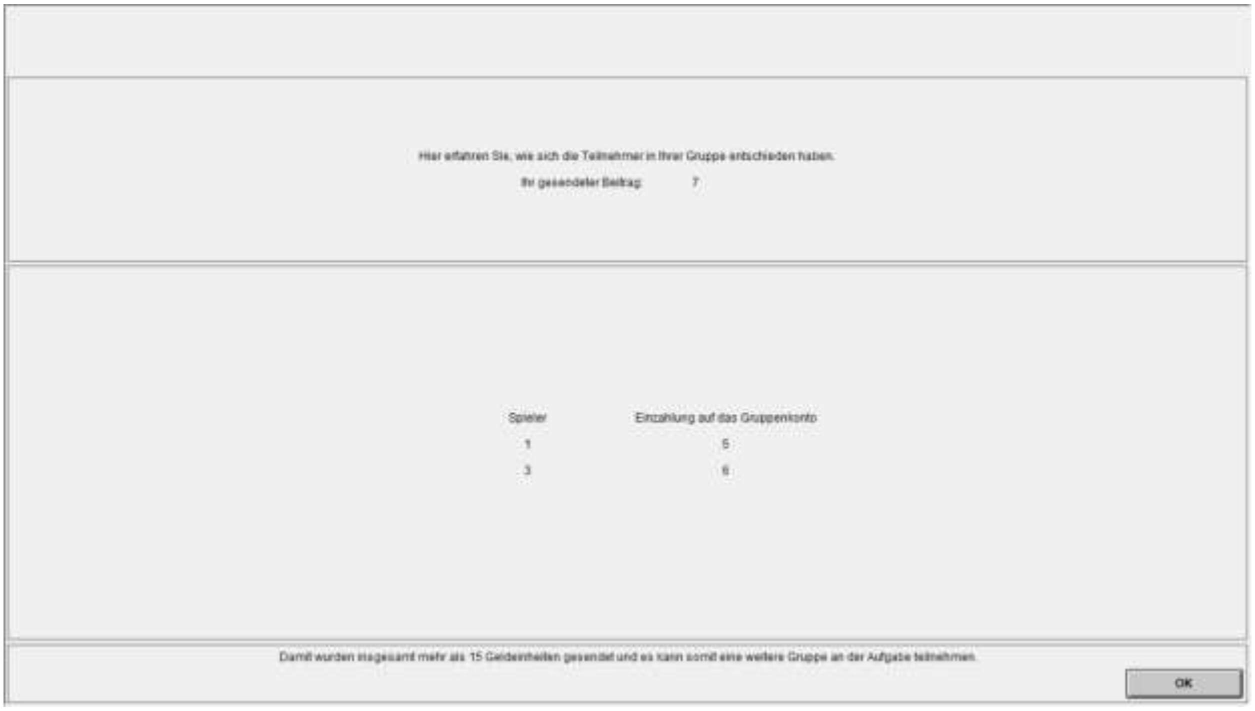

The second decision screen (The punishment screen __only available in punishment treatments__)

On this screen, you can make a decision if you want to assign negative points to another member. A negative point costs you 1 MU and reduces the earnings of the other participant in 3 MU. You can assign between 0 and 2 negative points to each participant

Player	Payment to the group account	Negative points
1	5	
3	6	

[Bottom right coroner "OK" button]

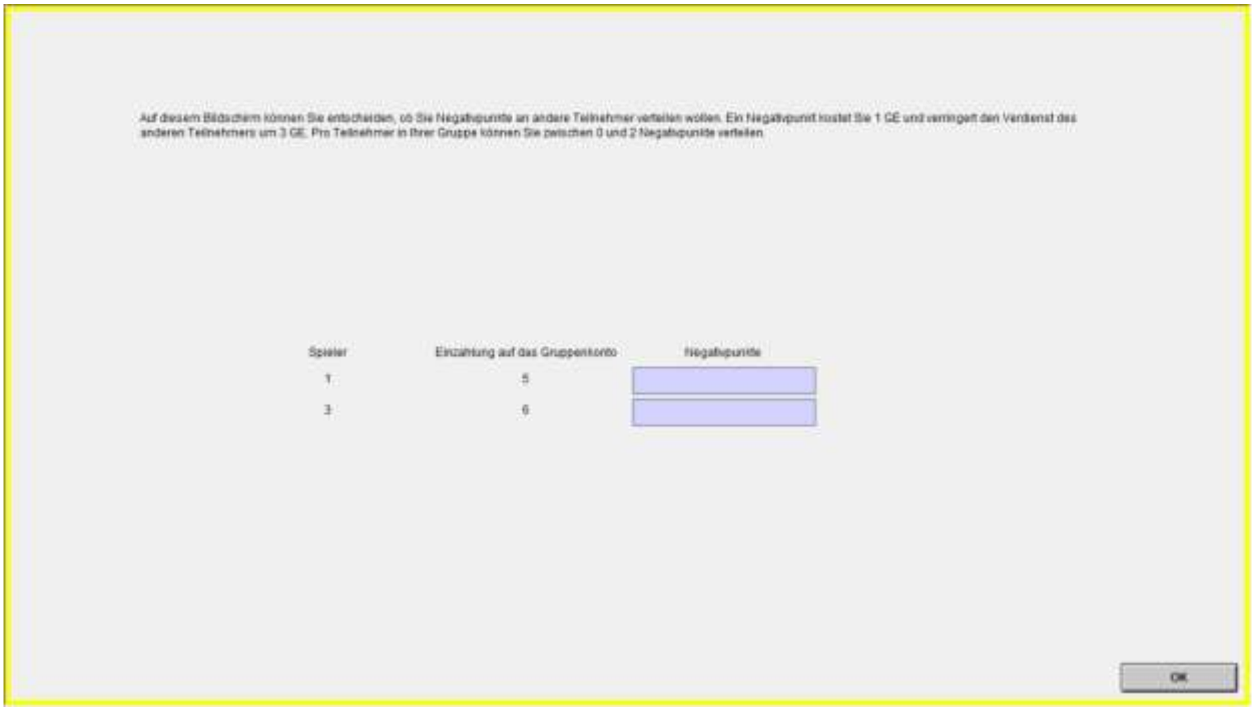

The second feedback screen

Here you are informed about the decisions of the other participants in your group

Your assigned endowment	7
Negative points received	3

[Bottom right coroner “OK” button]

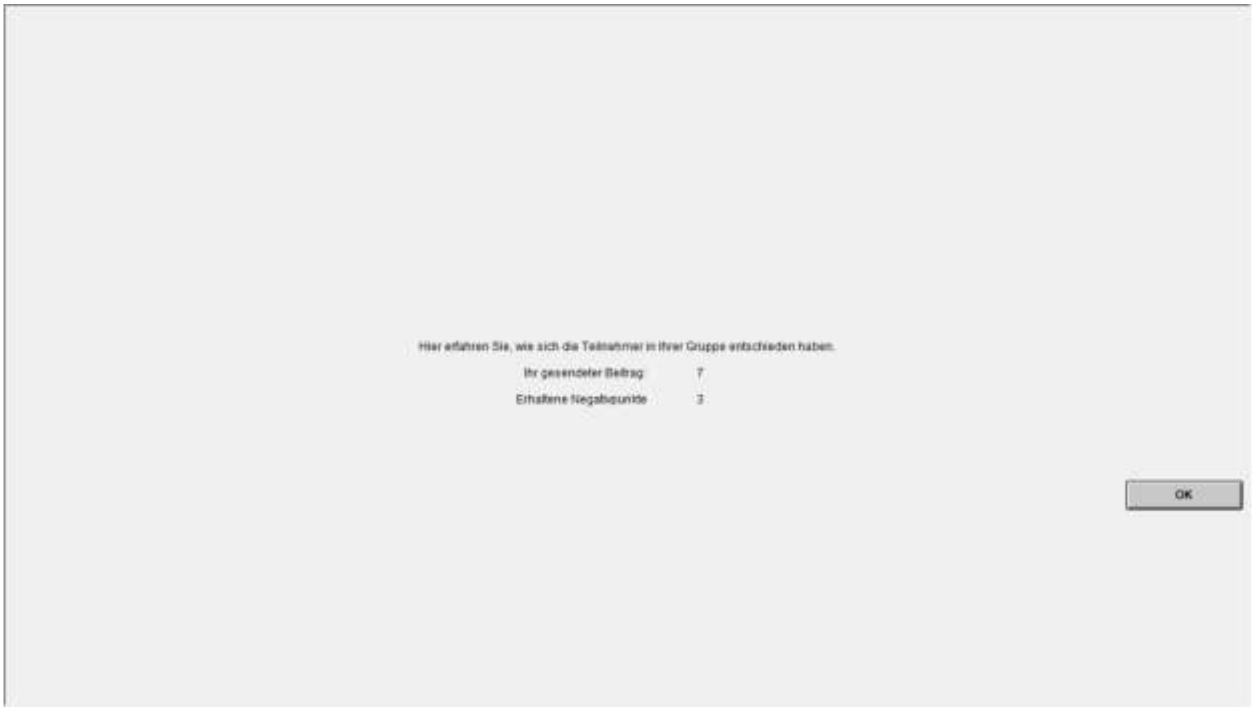

Supplementary References

1. Hauser, O. P., Rand, D. G., Peysakhovich, A., and Nowak, M. A. (2014). Cooperating with the future. *Nature*, 511(7508), 220.
