# Supplementary material for: The effects of contemporaneous peer punishment on cooperation with the future
Source: Nat Commun. 2020 Apr 14;11:1815. doi: 10.1038/s41467-020-15661-7 (PMC7156437; doi:10.1038/s41467-020-15661-7)
Supplement: Supplementary file 2 — Reporting Summary [file 41467_2020_15661_MOESM2_ESM.pdf]

nature research

Corresponding author(s): Israel Waichman

Last updated by author(s): Mar 16, 2020

Reporting Summary

Nature Research wishes to improve the reproducibility of the work that we publish. This form provides structure for consistency and transparency in reporting. For further information on Nature Research policies, see Authors & Referees and the Editorial Policy Checklist.

Statistics

For all statistical analyses, confirm that the following items are present in the figure legend, table legend, main text, or Methods section.

n/a	Confirmed	
☐	☒	The exact sample size (*n*) for each experimental group/condition, given as a discrete number and unit of measurement
☐	☒	A statement on whether measurements were taken from distinct samples or whether the same sample was measured repeatedly
☐	☒	The statistical test(s) used AND whether they are one- or two-sided<br>*Only common tests should be described solely by name; describe more complex techniques in the Methods section.*
☐	☒	A description of all covariates tested
☐	☒	A description of any assumptions or corrections, such as tests of normality and adjustment for multiple comparisons
☐	☒	A full description of the statistical parameters including central tendency (e.g. means) or other basic estimates (e.g. regression coefficient) AND variation (e.g. standard deviation) or associated estimates of uncertainty (e.g. confidence intervals)
☐	☒	For null hypothesis testing, the test statistic (e.g. *F*, *t*, *r*) with confidence intervals, effect sizes, degrees of freedom and *P* value noted<br>*Give P values as exact values whenever suitable.*
☒	☐	For Bayesian analysis, information on the choice of priors and Markov chain Monte Carlo settings
☒	☐	For hierarchical and complex designs, identification of the appropriate level for tests and full reporting of outcomes
☒	☐	Estimates of effect sizes (e.g. Cohen's *d*, Pearson's *r*), indicating how they were calculated

Our web collection on statistics for biologists contains articles on many of the points above.

Software and code

Policy information about availability of computer code

Data collection	ztree Version 3.6
Data analysis	Stata 15

For manuscripts utilizing custom algorithms or software that are central to the research but not yet described in published literature, software must be made available to editors/reviewers. We strongly encourage code deposition in a community repository (e.g. GitHub). See the Nature Research guidelines for submitting code & software for further information.

Data

Policy information about availability of data

All manuscripts must include a data availability statement. This statement should provide the following information, where applicable:

- Accession codes, unique identifiers, or web links for publicly available datasets
- A list of figures that have associated raw data
- A description of any restrictions on data availability

The experimental data that support the findings of this study are available in figshare with the identifier https://figshare.com/s/f140d450cb6758375da7

Field-specific reporting

Please select the one below that is the best fit for your research. If you are not sure, read the appropriate sections before making your selection.

☐ Life sciences ☒ Behavioural & social sciences ☐ Ecological, evolutionary & environmental sciences

Behavioural & social sciences study design

All studies must disclose on these points even when the disclosure is negative.

Study description	Quantitative study, lab experiment
Research sample	University students at two large German universities. Sample provides insights into a general causal mechanism according to the standards of the discipline.
Sampling strategy	Students participants were invited from an existing database of volunteer participants using an invitation software (Hroot). Participants were randomly allocated to baseline and treatment sessions. Sample size considerations were made based on the original findings of Hauser et al. so that we would be able to detect a treatment effect of similar size compared to their treatments at conventional levels of statistical significance and power.
Data collection	Data were collected via z-Tree experimental software in two different experimental economics labs. Sessions were conducted by the same set of two research assistants in each lab respectively. Research assistants were blind to the hypothesis to be tested and the treatment conditions conducted. Experimenters were only present for payment procedures.
Timing	We conducted a total of 35 sessions with 12 participants each for a total of 420 student participants from January to August of 2017.
Data exclusions	No data were excluded
Non-participation	No participants dropped out of the experiment after signing up for the study.
Randomization	participants were randomized to treatment conditions

Reporting for specific materials, systems and methods

We require information from authors about some types of materials, experimental systems and methods used in many studies. Here, indicate whether each material, system or method listed is relevant to your study. If you are not sure if a list item applies to your research, read the appropriate section before selecting a response.

Materials & experimental systems

n/a	Involved in the study	
☒	☐	Antibodies
☒	☐	Eukaryotic cell lines
☒	☐	Palaeontology
☒	☐	Animals and other organisms
☐	☒	Human research participants
☒	☐	Clinical data

Methods

n/a	Involved in the study	
☒	☐	ChIP-seq
☒	☐	Flow cytometry
☒	☐	MRI-based neuroimaging

Human research participants

Policy information about studies involving human research participants

Population characteristics	Student participants from the general participant pool of voluntary participants for economic experiments at the Universities of Heidelberg and Hamburg. Demographic information is only available for a subset of 96 participants as this information does not pertain to the hypotheses tested. (Avg. Age: 23.5; Avg. Gender: 56% female; mixed disciplines in Bachelor and Master programs)
Recruitment	Student participants from the general participant pool of voluntary participants for economic experiments at the Universities of Heidelberg and Hamburg. The recruitment materials did not reveal any specific details of the task or the hypotheses to be tested ruling out selection effects based on study content. As in any research that relies on voluntary participation the characteristics of participants reflect those of voluntary participants rather than a more general population. Our results are closely in line with those from a sample including non-university students. It is impossible to conduct research such as the one we describe with samples including non-volunteers.
Ethics oversight	University of Heidelberg

Note that full information on the approval of the study protocol must also be provided in the manuscript.
